# High-Yield Vanillin Production Through RSM-Optimized Solid-State Fermentation Process from Brewer’s Spent Grains in a Single-Use Bag Bioreactor

**DOI:** 10.3390/molecules30173452

**Published:** 2025-08-22

**Authors:** Ewa Szczepańska, Witold Pietrzak, Filip Boratyński

**Affiliations:** 1 Department of Food Chemistry and Biocatalysis, Wrocław University of Environmental and Life Sciences, Norwida 25, 50-375 Wrocław, Poland; filip.boratynski@upwr.edu.pl; 2 Department of Fermentation and Cereals Technology, Wrocław University of Environmental and Life Sciences, Chełmońskiego 37, 51-630 Wrocław, Poland; witold.pietrzak@upwr.edu.pl

**Keywords:** vanillin, solid-state fermentation, biosynthesis, by-products, optimization, RSM

## Abstract

Vanillin is the compound of great interest to the industry. It is used to augment and enhance the aroma and taste of food preparations and also as a fragrance compound in perfumes and detergents. Currently, majority of the world’s supply consists of chemically synthesized or lignin-derived vanillin. The application of biocatalysis for sustainable manufacturing of food ingredients, pharmaceutical intermediates, and fine chemicals is the key concept of modern industrial biotechnology. The main goal of this research was to conduct optimization procedures aimed at intensifying the microbial hydrolysis process of the lignin-rich plant raw materials and further bioconversion of the released ferulic acid to vanillin. The tests were performed in the solid-state fermentation system with strains selected during the screening stage on agri-food by-products such as brewer’s spent grain. A specially designed single-use bag bioreactor was used to carry out the process on a preparative scale with the most effective strain. The experiment was designed using the RSM, which allowed for an increase in biosynthesis efficiency from 363 mg/kg to 1413 mg/kg (an increase of 389%). The progress of the process was controlled by the use of chromatographic techniques (HPLC) by quantitative determination of vanillin content in the obtained extracts.

## 1. Introduction

Vanillin (4-hydroxy-3-methoxybenzaldehyde) is a phenolic compound found mainly in the tropical vanilla plant *Vanilla planifolia*. Vanillin is widely used as the flavoring and fragrance ingredient in the food and cosmetic industries. This compound also exhibits a wide range of biological properties, which is used in the pharmaceutical industry [1]. In the global market, less than 1% of vanillin is extracted from vanilla pods, while the remaining percentage is synthetic and lignin-derived vanillin [2]. The price of natural vanillin stands high compared to its synthetic equivalent, which is mainly due to the limited availability of vanilla pods and yield fluctuations related to climatic conditions. Chemically synthesized vanillin is an artificial aromatic compound produced from fossil hydrocarbons. Approximately 85% of commercially available vanillin is produced by this method [3]. Chemical synthesis of vanillin presents significant environmental and sustainability challenges. While this approach is economically efficient and satisfies the high global demand for vanillin, its reliance on non-renewable resources and its generation of hazardous waste are major concerns. The chemical synthesis of vanillin often involves harsh reaction conditions and the use of hazardous chemicals, including strong oxidizing agents and organic solvents like toluene and ethyl acetate [4]. The transition to more sustainable production methods is crucial for addressing the environmental impact and meeting the growing consumer demand for natural products. Therefore, more and more attention is being paid to limiting the use of compounds obtained by chemical methods and replacing them with more natural equivalents. This has led to an increased interest among researchers in exploring alternative methods of producing vanillin.

Biotechnological methods for vanillin production offer significant advantages over traditional chemical synthesis, addressing major environmental, economic, and market-driven concerns, as they align with the principles of a circular bioeconomy, reducing waste, and decreasing reliance on fossil fuels. These processes are characterized primarily by the mild conditions of the processes, and the resulting product’s classification. Therefore, various biotechnological approaches have been proposed, including enzymatic and microbiological procedures using a natural substrate to obtain vanillin, which can be classified as “naturally identical” in accordance with European Union regulations [5]. Vanillin precursors include sugars and phenolic compounds such as isoeugenol, eugenol, ferulic acid, vanillic acid, and aromatic amino acids. Due to the similarity of structures, ferulic acid is one of the most popular precursors used for vanillin biosynthesis. Ferulic acid is a hydroxycinnamic acid, abundant in natural sources, forming cross-links with lignin and polysaccharides, which harden the cell wall [6]. Sources include waste and by-products of the agri-food industry such as wheat straw, rice and oat husks, wheat bran, and corn cobs which contain a significant amount of lignin, favoring the production of vanillin. The agri-food industry generates waste and by-products in large quantities worldwide, which causes problems with their disposal. These wastes are a rich source of sugars, proteins, fats, and some other important precursors for the production of value-added compounds. At the same time, they are sustainable and inexpensive raw materials, and their valorization by appropriate sustainable approaches to value-added compounds is emerging as a strong research trend [2]. Nowadays, enzymatic methods and microbial fermentations are increasingly used as methods for obtaining vanillin. However, researchers mainly focus on the use of microbial fermentation as an environmentally friendly and cost-effective approach [7].

The literature describes mainly two methods of microbiological synthesis of vanillin using waste and by-products of the agri-food industry. The first one introduces ferulic acid being released and extracted from lignocellulosic waste into microbiological cultures as a substrate [8,9]. The second approach consists of carrying out microbiological culture in a liquid medium, which is usually the source of carbon and at the same time the precursor is lignocellulosic plant biomass [10,11]. There are also reports on the use of the method of culturing on a solid medium (solid-state fermentation, SSF) [12]. According to the concept, this process is performed at low water activity, and the assumption is to use a solid raw material as a substrate, which is a source of essential nutrients and building blocks for microbial cells. The main group of microorganisms that have evolved mechanisms to transform lignocellulose under solid-state fermentation conditions are fungi, which offer a new and more economical route to produce valuable compounds. Due to the solid nature of the substrate, it is difficult to monitor environmental parameters such as pH, aeration, humidity, and the amount of accumulated biomass. However, with the development of measurement and optimization techniques, these limitations can be eliminated [2]. SSF has been extended as a new exemplary bioconversion approach for vanillin production.

Currently, it is crucial to optimize developed processes to maximize their efficiency. Mathematical and statistical techniques are used for this purpose, including one of the most well-known response surface method (RSM). The main objective of RSM is to optimize multiple reaction parameters to obtain higher yield and a purified final product at a lower cost. In the optimization process, a series of experiments are carried out covering all the key parameters such as incubation temperature, aeration conditions, incubation duration, and ratios of different reagents used. RSM has been applied to optimize the key reaction parameters during the bioconversion process for the production of vanillin and has shown satisfactory results [8,10,12].

This paper reports the optimization procedures aimed at intensifying the microbial hydrolysis process of the lignin-rich plant raw materials and the further bioconversion of the released ferulic acid to vanillin. The tests were performed in a solid-state fermentation culture system using fungal strains selected during the screening stage on agri-food industry by-products such as brewer’s spent grain. The experiment was designed using the RSM, during which a matrix was designed, taking into account three levels of individual variables (−1, 0, +1), such as substrate moisture, cultivation temperature, degree of fragmentation of the substrate, and optical density of the inoculum. The last part of the research was conducted in the preparative scale using the specially designed single-use bag bioreactor. The three levels of the factors such as temperature, time, and air flow were considered. The mathematical model was appointed in the experimental environment of the DesignExpert v13 software.

## 2. Results and Discussion

### 2.1. Screening Scale of Solid-State Fermentation

The first stage of the research on the biosynthesis of vanillin by solid-state fermentation system consisted of screening 150 various strains of microorganism on two agri-food industry by-products such as Brewer’s Spent Grain (BSG) and Linseed Oil Cake (LOC) (see Supplementary Materials, Tables S1–S3). The criteria for selecting raw materials were the prevalence of the by-products in Poland and a high content of lignocellulosic fractions. Among the tested strains, seven of them were selected as biocatalysts that were able to produce vanillin (Table 1), and the most suitable raw material was BSG. The next step of the experiment was the maximization of the vanillin biosynthesis by the optimization of the process parameters. The statistical optimization was performed in Erlenmeyer flasks through the Box–Behnken design of RSM by checking the effect of different parameters like moisture content (50 to 70%), temperature (25–35 °C), degree of particles fragmentation (ø 3.5 mm, 2 mm, 0.5 mm), and optical density of spores suspension used for the inoculation of raw materials (OD_600_ 0.2–0.4). The incubation lasted 6 days, which was confirmed as an optimal time during the screening process. The DesignExpert v13 software generated 29 trials including three levels of four factors. The results were calculated by giving the amount of vanillin in the obtained extracts per 1 kg of dry mass of raw material and are presented in the table below (full version of Table 1 in Supplementary Materials, Table S4).

The highest amounts of vanillin were recovered from the extracts from *Phanerochaete chrysosporium* CBS246.84 culture (363 mg of vanillin/kg of dry mass of raw material) and *P. chrysosporium* CBS481.73 (229 mg of vanillin/kg of dry mass of raw material) (Run no. 5, Table 1). All the tested strains belonging to the *P. chrysosporium* species exhibited the highest ability for vanillin biosynthesis in the same conditions (60% moisture, 30 °C, OD_600_ = 0.4). Based on the ANOVA results generated for all the tested strains, model F-value was significant, and the lack of fit F-value implied that the lack of fit was not significant relative to the pure error. Moreover, all the ANOVA results indicated that the degree of particle fragmentation significantly influenced the vanillin yield (*p*-value < 0.0001). It is likely that the high level of fragmentation allowed better access to both the nutrients and the vanillin precursor available in the raw material. The optimal level of substrate moisture was 60% for most of the strains tested, except *Aspergillus flavus* KKP3556, where higher vanillin content in the extract was determined in the culture carried out at 70% water content. It was also observed that for Ascomycota fungi (*Aspergillus*, *Fusarium*) the optimal level of spore solution optical density was 0.3; however, the p-value for this factor indicated that it was not significant for the model.

Brewer’s Spent Grain (BSG) is a significant by-product of the brewing industry, constituting approximately 80% of all by-products produced. It consists mainly of the seed pericarp and husk layers of barley (*Hordeum vulgare*) as well as the insoluble remnants from other ingredients used in beer-making, including both raw and malted cereals. Each year, the global production of BSG is about 39 million tons, with Europe contributing about 10% of that total. On a dry weight basis, roughly half of BSG is made up of fiber, predominantly hemicellulose and cellulose. Additionally, BSG contains substantial amounts of proteins, which can make up to 30% of its overall composition, along with materials rich in phenolics [13]. Lignin, a polyphenolic macromolecule, is found at up to 28% BSG and consists of not only monomers sinapyl/coniferyl and p-coumaroyl alcohol but also high amounts of phenolic compounds that are also vanillin precursors, such as ferulic acid or vanillic acid [14,15].

As is reported in the literature, microorganisms such as white-rot fungi (*Phanerochaete*, *Pycnoporus*, *Aspergillus*) have the ability to break down lignin into smaller aromatic compounds. Enzymes like laccases and peroxidases (e.g., lignin peroxidase, manganese peroxidase), have an essential role in this process [16,17,18,19,20]. Following depolymerization, these smaller aromatic units undergo further enzymatic degradation and oxidation. This oxidative breakdown results in intermediate compounds like ferulic acid and vanillic acid. The final stage is the conversion of the above-mentioned compound into vanillin. Fungi are capable of efficiently carrying out these steps, making the microbial conversion of lignin to vanillin an effective and sustainable method for producing this valuable compound [21,22,23,24,25].

It is worth mentioning that even with the careful maintenance and management of fungal biomass in engineered processes, the stability of its ligninolytic activity can be unpredictable. This activity, part of the organism’s secondary metabolism, is influenced by a complex array of nutritional, physiological, and environmental factors. It involves the production of various intracellular and extracellular enzymes, along with redox-mediating metabolites, yet the precise mechanisms and interactions involved remain largely unclear. As a result, operational challenges and gaps in knowledge have hindered the widespread application of fungal bioprocesses [16,18,26]. Additionally, in this research, the process involves the disposal of organic by-products such as BSG, the composition and properties of which are not constant and may change. This may be influenced by factors related to the origin of the raw material, such as the variety of barley grains, harvesting time, addition of adjuncts, brewing technology, and hop composition [27]. Factors influencing process performance should be identified and standardized to maintain process stability.

### 2.2. Preparative Scale of Solid-State Fermentation

The second stage of the experiment was performed at a larger scale (using 300 g of the raw material) in the single-use bag bioreactor, the general diagram of which is shown in Section 3.4. The bioreactor chamber was constructed using a polyamide-6 sterilization foil sleeve. This material met the criteria for tightness, flexibility, and autoclave resistance. The bioreactor design was chosen based on the need for easy scalability, rapid preparation, and elimination of the need for large autoclaves. The application of a single-use bag system minimizes the risk of cross-contamination between batches and ensures the sterility of the process. There is no need for complex piping, cleaning systems, and extensive validation of cleaning processes, making it an attractive option for startups and pilot-scale production. However, it may not be suitable for the large-scale production of high-volume products. While the initial capital cost is low, the recurring cost of purchasing and disposing of single-use bags is high. This can make them more expensive on a long-term, large-scale basis compared to traditional bioreactors. The optimization process was conducted using *P. chrysosprium* CBS246.84, selected as the most efficient strain in screening scale. The 17 trials were generated using the DesignExpert v13 software (Box–Behnken design) containing three levels of three factors such as temperature, air flow, and time. The statistical analyses were performed to examine their individual and combined effects on vanillin synthesis.

The maximum vanillin (1321.8 mg/kg of dry mass of BSG) production was achieved after 6 days at 27 °C and air flow equal to 1.75 nL/min, while the minimum production (257.6 mg/kg) was observed after 4 days at 22 °C and air flow equal to 1.75 nL/min as summarized in Table 2. The analysis of variance (ANOVA) for vanillin production represents that the suggested model is significant as indicated by the *p*-value (<0.0001). The statistical significance of the model, as indicated by the F-value is observed as 80.31. The lack of fit of F-value of the model is 5.49, which is not significant and may be considered as pure error (Table 3). The coefficient of determinant (R^2^) that indicates the variability of the model and real relationship between variables is found to be 0.9904. Predicted R^2^ (0.8736) is in reasonable agreement with the adjusted R^2^ (0.9781).

**Figure 1:**
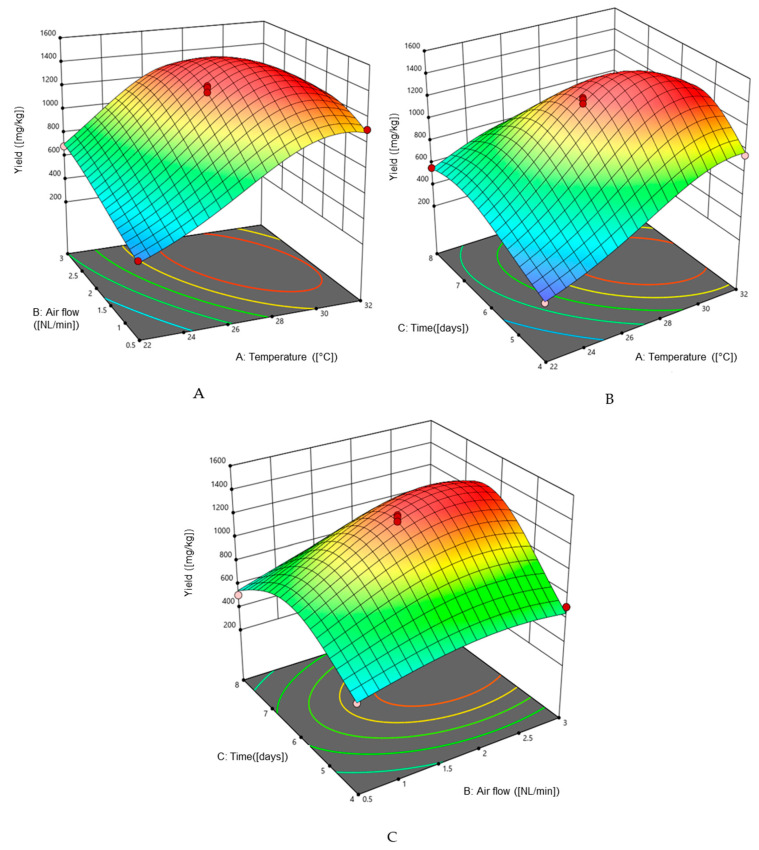
Response surface graph of vanillin biosynthesis by *P. chrysosporium* CBS246.84 in the single-use bag bioreactor. The effect of different factors was represented as (**A**) air flow and temperature, (**B**) time and temperature, (**C**) time and air flow.

From the ANOVA (Table 3), it is determined that the effects of all factors are significant. The relationships between them are shown in Figure 1. The transformation of the dependent variable y was performed, the analysis indicated that the best model would be the inverse square root model. The interaction of variable parameters like temperature, air flow, and incubation time are indicated by the mathematical formula:

Vanillin = 0.541630 − 0.023848 × Temperature − 0.030516 × Air flow − 0.046668 × Time  + 0.000811 × Temperature × Air flow + 0.000735 × Temperature × Time − 0.000272 × Air flow × Time + 0.000307 × Temperature^2^ + 0.002116 × Air flow^2^ + 0.002195 × Time^2^

A validation of the model was achieved by performing the experiment in the bioreactor considering the value of factors suggested by the DesignExpert software as the most optimal (temperature 28 °C, air flow 2.25 nL/min, incubation time 6 days). The predicted response for vanillin production was 1428.32 mg/kg, and an actual response was 1413.32 mg/kg, which proved model validity.

Agri-food by-products are sustainable and inexpensive raw materials, rich in important precursors for the production of bioactive compounds such as vanillin. Therefore, their transformation into value-added compounds using appropriate sustainable methods is emerging as a strong research trend. The research on the use of lignocellulosic by-products mainly focuses on the chemical and microbiological hydrolysis, and the isolation of ferulic acid, which was then used as a substrate for vanillin biosynthesis [8,9,28,29,30]. Another trend in the use of agri-industrial residues is their addition to liquid media as a direct source of vanillin precursor. Chattopadhyay et al. conducted research on the biosynthesis of vanillin from wheat bran using *Streptomyces sannanensis* MTCC 6637 strain. Additionally, optimization was performed by central composite design (CCD) of RSM. The optimum vanillin production (708 mg/L) was achieved after 5 days of incubation in the medium containing de-starched wheat bran (10% *w*/*v*), sucrose (0.2% *w*/*v*), peptone (1% *w*/*v*) at pH 7.5, agitation 220 rpm, and temperature 28 °C [10]. Sugarcane bagasse was used as the raw material for obtaining phenolic compounds such as ferulic acid, vanillic acid, and vanillin, with *Lactobacillus acidophilus* MTCC 10307 as the biocatalyst. The highest concentration of vanillin was detected in the post-fermentation medium after the 12th day of incubation (15 mg/mL of extract) [11].

The SSF method has been proposed as a new method for the bioconversion of lignocellulosic by-products to vanillin as it offers a higher yield and production efficiency and better product properties than submerged fermentation. The study conducted by Nurika et al. demonstrated the potential of using rice straw to obtain vanillin. The experiments were conducted using solid-state fermentation in honey jars containing 10 g of rice straw and water inoculated with *Serpula lacrymans*. After 35 days, a mixture of high value bio-based compounds, including vanillin at the amount of 3957 mg/kg of raw material was obtained [31]. Various agricultural lignocellulosic by-products (sugarcane bagasse, wheat straw, rice straw, rice bran, and corn cob) were tested for biotransformation into vanillin by Mehmood et al. using SSF. Tested raw materials were introduced in an Erlenmeyer flask, where the substrate: water ratio was established at 1:3 using the sterilized basal medium and inoculated with *Enterobacter hormaechei* then incubated for 48 h at 30 °C. Among agricultural by-products tested, sugarcane bagasse proved to be the raw material of choice and as a result 290 mg/kg of vanillin was obtained. Then, different physicochemical parameters such as moisture content, temperature, pH, inoculum size, and incubation time, were optimized using CCD of response surface methodology. After optimization, the highest concentration of vanillin (4760 mg/kg of sugarcane bagasse) was achieved at a moisture content of 70%, temperature of 37.5 °C, pH of 7.5, inoculum size of 4 mL and incubation time of 48 h [32]. Wheat straw was used as a source of ferulic acid for vanillin synthesis using *Streptomyces sannanensis* and SSF culture system by the Mehmood et al. research team. The experiments were conducted in the Erlenmeyer flasks (250 mL) containing 10 g of wheat straw. The moisture content varied from 40 to 80% (adjusted with the basal media), pH varied from 5 to 10, different inoculum volumes (1–5 mL) were optimized as well as incubation temperatures (25–50 °C), and the fermentation lasted from 8 to 120 h. The highest production of vanillin (2740 mg/kg) was observed at 70% moisture content, 72 h incubation time, 2 mL inoculum volume, 7.5 pH, and 35 °C [12]. It is worth emphasizing that the studies discussed above on the use of SSF for vanillin biosynthesis were conducted on a laboratory scale. This work included the first bench-scale studies in a bioreactor specifically designed for microbiological cultivation using agri-food industry by-products. This is a promising approach, as it not only incorporates a simple bioreactor design but also allows for easy scale-up. However, it is worth emphasizing that this is the first of many steps toward industrialization. The process requires a more detailed understanding of the mechanism and identification of factors that maximize the efficiency of vanillin biosynthesis. Undeniably, this is an interesting alternative to obtaining vanillin, potentially replacing its synthetic counterpart to some extent, but efforts are necessary to achieve economic viability.

## 3. Materials and Methods

### 3.1. Raw Materials and Chemicals

Vanillin and ferulic acid as reference compounds, and solvents used for extractions and HPLC analysis were purchased from Sigma-Aldrich (Darmstadt, Germany). Brewer’s Spent Grain (BSG) was obtained from the local brewery Złoty Pies (Wrocław, Poland).

Brewer’s Spent Grain was dried at 60 °C for 48 h to remove the moisture from it. After drying, it was ground into a fine powder by an electric grinder and sieved through the mesh size of 3.5 mm, 2 mm, and 0.5 mm. Then, it was used as a substrate for optimization experiments.

### 3.2. Microorganisms

*Aspergillus* sp. AM31 was purchased from the microbial collection of Department of Food Chemistry and Biocatalysis at Wrocław University of Environmental and Life Sciences (AM). *Phanerochaete chrysosporium* CBS246.84 and *Phanerochaete chrysosporium* CBS481.73 were purchased from Westerdijk Fungal Biodiversity Institute (CBS) in Utrecht (Netherlands). *Aspergillus flavus* KKP3556 was purchased from the Institute of Agricultural and Food Biotechnology State Research Institute Collection of Industrial Microorganisms (KKP) (Warsaw, Poland). *Pycnoporus cinnabarinus* DSM3022 and *Phanerochaete chrysosporium* DSM6909 were purchased from the German Collection of Microorganisms and Cell Cultures (DSMZ) in Braunschweig (DSMZ). *Fusarium culmorum* MUT5855 was obtained from Istituto di Scienze e Tecnologie Chimiche “Giulio Natta”-Consiglio Nazionale delle Ricerche (SCITEC-CNR) (Milan, Italy). Fungal strains were maintained at 4 °C on Czapek’s medium agar slants (g/L–sucrose 30, sodium nitrate 3, dipotassium phosphate 1, magnesium sulfate 0.5, potassium chloride 0.5, ferrous sulfate 0.001, agar 15).

### 3.3. Screening Scale of Solid-State Fermentation and Optimization of the Process Parameters by RSM

Cultures were carried out in 250 mL Erlenmayer flasks using 10 g of BSG with 3 sizes of particles fragmentation (ø 3.5 mm, 2 mm, 0.5 mm). After sterilization (121 °C, 15 min) the moisture content was adjusted from 50 to 70% with sterile deionized water containing spores suspension (OD_600_ 0.2–0.4) and incubated for 144 h at temperatures ranging from 25 to 35 °C. The 29 trials were generated containing four factors and the statistical analyses were performed using the DesignExpert v13 software (Box–Behnken design) considering 3 levels of factors variables to examine their individual and combined effects on vanillin yield (Table 4).

### 3.4. Preparative Scale of Solid-State Fermentation and Optimization of the Process Parameters by RSM

**Figure 2:**
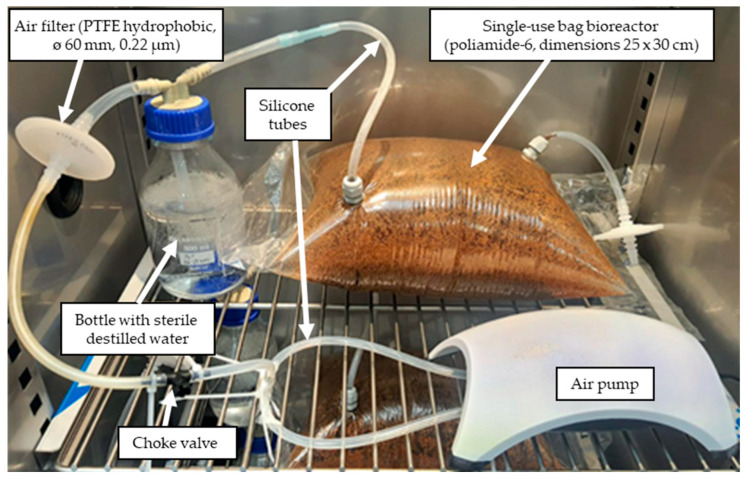
The single-use bag bioreactor used in the preparative stage of the experiment.

Cultures were performed in a single-use bag bioreactor designed for this research. The bioreactor chamber (dimensions 25 × 30 cm) was constructed using a polyamide-6 sterilization foil sleeve. Using a heat sealer, 300 g of BSG was sealed inside the chamber, and openings were created for cable glands, into which silicone air supply tubes were inserted. Additionally, the bioreactor includes a bottle of sterile distilled water through which filtrated air supplied by a pump flows (Figure 2). After sterilization (121 °C, 15 min) the moisture content of BSG was adjusted to 60% with sterile distilled water containing spores and incubated for 96, 144, and 196 h at temperatures ranging from 22 to 32 °C. To provide oxygen to the bioreactor, air sterilized by filtration (PTFE hydrophobic filters 60 mm, 0.22 µm) was introduced by aquarium air pumps through a silicone tube. Air flow rate was measured at the outlet of the bioreactor using a Kobold rotameter (model no. KFR-2114N0, Arnhem, the Netherlands) scaled in nL/min; to adjust the air flow, a choke valve was mounted on the air flow line. To prevent the solid substrate from drying out, the air was humidified by passing it through a bottle containing sterile distilled water.

The 17 trials were generated containing three factors and the statistical analyses were performed using the DesignExpert v13 software (Box–Behnken design) considering 3 levels of factors variables to examine their individual and combined effects on vanillin yield (Table 5).

### 3.5. Extraction Procedure

The entire culture was collected from the Erlenmayer flask for extraction and further analysis at individual time intervals. Fifteen mL of ethyl acetate was added to the post-culture medium, collected in a falcon (50 mL capacity), and shaken for 24 h at 1500 rpm (model Multi Reax 115V, Heidolph, Schwabach, Germany). After centrifugation (4000 rpm, 15 min), the organic fraction was separated and dried using anhydrous MgSO_4_. Samples evaporated under a stream of nitrogen were suspended in methanol, filtered using syringe filters into vials, intended for analysis by HPLC (UltiMate 3000 Dionex, Sunnyvale, CA, USA).

### 3.6. Preparative Scale Extraction Procedure

The extraction of culture from the single-use bag bioreactor system was performed with a different approach using centrifuge bottles (500 mL). The bioreactor content was extracted using methanol (the ratio of sample to solvent 1:10 *w*/*w*). Extraction was performed at room temperature on a shaker (220 rpm) for 1 h. After this time, samples were centrifuged (8000 rpm, 20 min) and after pouring off the extract, another portion of solvent was added, and the procedure was repeated (one sample was extracted 3 times to ensure complete extraction of vanillin). Collected extracts were evaporated by vacuum evaporator and placed in 100 mL volumetric flasks. Subsequently, 2 mL of sample was filtered using syringe filters into vials and analyzed by HPLC.

### 3.7. Analysis Procedure

Properly prepared samples were analyzed by chromatographic method under conditions selected for the determination of vanillin. Samples were examined by HPLC using an UltiMate 3000 instrument (Dionex, Sunnyvale, CA, USA) with a UV detector, using a C18 packed column (25 cm × 4.6 mm, 5 μm). The mobile phase consisted of aqueous 0.5% formic acid (solution A) and methanol (solution B) flow 1 mL/min. A/B (*v*/*v*): 0–3 min (70:30), 11 min (25:75), 13 min (0:100), 21 min (70:30). Absorbance was measured at 281 nm and 254 nm. For quantitative analysis of vanillin, a standard curve was prepared and its content in kg of dry matter of the by-product was calculated.

## 4. Conclusions

The economic and ecological utilization of lignocellulosic by-products remains a significant challenge. Microbial cell factories offer a promising and sustainable approach to their bioconversion. By utilizing diverse fungal strains equipped with lignin-degrading enzymes, this process can efficiently convert lignin into valuable aromatic compounds, including vanillin. In this study, brewer’s spent grain was used as a source of ferulic acid as one of the most easily available lignocellulosic by-products in Poland. Different parameters of the solid-state fermentation process were optimized through a Box–Behnken design of response surface methodology. The maximum production of vanillin (1413.32 mg/kg) was achieved at 60% moisture content, temperature 28 °C, air flow 2.25 nL/min, and incubation time of 6 days. This is the first large-scale study of the application of the SSF culture system for vanillin biosynthesis. A single-use bag bioreactor was used for this purpose, which is characterized by a simple design and the possibility of further scaling up of production and implementation of the process at a pilot scale. This is the first step towards implementing industrial-scale production using a bioreactor adapted for this purpose. Further work is necessary to increase the cost-effectiveness of this process, including a more in-depth understanding of the mechanism and factors influencing efficiency of vanillin synthesis. Continued advances have the potential to increase the cost-effectiveness of microbial vanillin production, providing an eco-friendly alternative to traditional chemical methods.

## Figures and Tables

**Table 1:** Selected results of optimization process through the Box–Behnken design on vanillin biosynthesis in Erlenmeyer flasks from BSG.

Run No.	A	B	C	D	Vanillin Content in the Extracts [mg/kg d.m. of Substrate]						
					*Aspergillus* sp. AM31	*P. chrysosporium* CBS246.84	*P. chrysosporium* CBS481.73	*A. flavus* KKP3556	*P. cinnabarinus* DSM3022	*P. chrysosporium* DSM6909	*F. culmorum* MUT5855
2	60	35	0.5	0.3	70	221	172	137	**204**	132	134
5	60	30	0.5	0.4	95	**363**	**229**	123	114	**164**	186
6	70	30	0.5	0.3	121	194	183	**218**	139	148	103
23	60	25	0.5	0.3	**176**	298	189	129	85	139	**203**

A—moisture content (%); B—temperature (°C); C—degree of particles fragmentation (mm); D—OD_600_ of spores suspension.

**Table 2:** Results of optimization process through the Box–Behnken design on vanillin biosynthesis from BSG in the single-use bag bioreactor.

Run No.	A	B	C	Vanillin Content in the Extracts [mg/kg d.m. of Substrate]
1	32	1.75	4	957
2	22	1.75	8	559.7
3	22	0.5	6	346.6
4	27	1.75	6	1321.8
5	27	0.5	4	490.5
6	32	1.75	8	575.2
7	27	1.75	6	1268.9
8	32	0.5	6	1107.9
9	27	1.75	6	1269.6
10	27	1.75	6	1137
11	32	3	6	827
12	27	3	4	704.2
13	27	1.75	6	1306.8
14	27	0.5	8	511
15	22	3	6	686.3
16	27	3	8	862.4
17	22	1.75	4	257.6

A—temperature (°C), B—air flow (nL/min), C—time (days).

**Table 3:** ANOVA for factors affecting vanillin synthesis from BSG in the single-use bag bioreactor.

Source	Sum of Squares	df ^1^	Mean Square	F-Value	*p*-Value	
Model	0.0015	9	0.0002	80.31	<0.0001	significant
A—Temperature	0.0004	1	0.0004	194.06	<0.0001	
B—Air flow	0.0001	1	0.0001	47.36	0.0002	
C—Time	0.0000	1	0.0000	13.52	0.0079	
AB	0.0001	1	0.0001	48.03	0.0002	
AC	0.0002	1	0.0002	101.08	<0.0001	
BC	1.844 × 10^−6^	1	1.844 × 10^−6^	0.8621	0.3840	
A^2^	0.0002	1	0.0002	115.98	<0.0001	
B^2^	0.0000	1	0.0000	21.53	0.0024	
C^2^	0.0003	1	0.0003	151.85	<0.0001	
Residual	0.0000	7	2.138 × 10^−6^			
Lack of fit	0.0000	3	4.015 × 10^−6^	5.49	0.0667	not significant
Pure error	2.925 × 10^−6^	4	7.313 × 10^−7^			
Cor Total	0.0016	16				

^1^ df—degree of freedom.

**Table 4:** Box–Behnken design for optimization of vanillin synthesis from BSG.

Run No.	A	B	C	D
1	60	35	2	0.2
2	60	35	0.5	0.3
3	70	25	2	0.3
4	60	30	2	0.3
5	60	30	0.5	0.4
6	70	30	0.5	0.3
7	60	30	0.5	0.2
8	50	35	2	0.3
9	60	25	2	0.4
10	60	30	2	0.3
11	60	35	3.5	0.3
12	60	30	3.5	0.2
13	60	25	2	0.2
14	70	35	2	0.3
15	70	30	3.5	0.3
16	50	30	2	0.4
17	60	25	3.5	0.3
18	50	25	2	0.3
19	70	30	2	0.2
20	70	30	2	0.4
21	60	35	2	0.4
22	50	30	0.5	0.3
23	60	25	0.5	0.3
24	60	30	2	0.3
25	60	30	3.5	0.4
26	60	30	2	0.3
27	50	30	3.5	0.3
28	50	30	2	0.2
29	60	30	2	0.3

A—moisture content, B—temperature, C—degree of particles fragmentation, D—OD_600_ of spores suspension.

**Table 5:** Box–Behnken design for optimization of vanillin synthesis from BSG in the single-use bag bioreactor.

Run No.	A	B	C
1	32	1.75	4
2	22	1.75	8
3	22	0.5	6
4	27	1.75	6
5	27	0.5	4
6	32	1.75	8
7	27	1.75	6
8	32	0.5	6
9	27	1.75	6
10	27	1.75	6
11	32	3	6
12	27	3	4
13	27	1.75	6
14	27	0.5	8
15	22	3	6
16	27	3	8
17	22	1.75	4

A—temperature (°C), B—air flow (nL/min), C—time (days).

## Data Availability

The data are contained within the article; further inquiries can be directed to the corresponding authors.

## Supplementary Materials

The following supporting information can be downloaded at https://www.mdpi.com/article/10.3390/molecules30173452/s1, Table S1: Strains from collection of the Department of Food Chemistry and Biocatalysis at Wrocław University of Environmental and Life Sciences, Table S2: Strains purchased from the ATCC, KKP, DSMZ, and CBS collection, Table S3: Strains isolated from various environments, Table S4: Results of optimization process through the Box–Behnken design on vanillin biosynthesis in Erlenmeyer flasks from BSG.
