# Supplementary material for: High-Yield Vanillin Production Through RSM-Optimized Solid-State Fermentation Process from Brewer’s Spent Grains in a Single-Use Bag Bioreactor
Source: Molecules. 2025 Aug 22;30(17):3452. doi: 10.3390/molecules30173452 (PMC12430523; doi:10.3390/molecules30173452)
Supplement: Supplementary file 1 [file molecules-30-03452-s001.zip › molecules-3822573-supplementary.pdf]

1. Strains used for the screening process (Table S1-S3):

• The strains came from the strain collection of the Department of Food Chemistry and Biocatalysis at Wrocław University of Environmental and Life Sciences (Table S1).
• The strains were purchased from the PCM, DSMZ, KKP, ATCC, and CBS collections (Table S2).
• The strains were isolated from various environments (Table S3). Samples were collected during fieldwork in sterile bags and then subjected to mycological analysis. Various isolation methods were used to obtain pure fungal strains from the environment for screening. Most strains were isolated from decaying wood tissues due to their ability to degrade lignocellulosic materials, according to methodology proposed in the literature. Some strains were isolated from fungal fruiting bodies. In this method, small pieces (3x3mm) of mycelium obtained from the inner part of the mushroom (to avoid cross-contamination) were placed on PDA medium. Each method involved passage of growing fungal colonies, followed by monospore culture to obtain pure mycelium.

Table S1. Strains from collection of the Department of Food Chemistry and Biocatalysis at Wrocław University of Environmental and Life Sciences.

No.	Strain name	Strain code
1	*Fusarium culmorum*	MUT 5855
2	*Streptomyces xantochromogenes*	IPV 2641
3	*Fusarium acuminatum*	S1
4	*Penicillium chrysogenum*	S4
5	*Mucor hiemalis*	W2
6	*Penicillium commune*	W7
7	*Aspergillus versicolor*	TJ1
8	*Trichoderma atroviride*	TRW
9	*Fusarium culmorum*	AM7
10	*Fusarium culmorum*	AM9
11	*Fusarium culmorum*	AM10
12	*Fusarium avenaceum*	AM11
13	*Fusarium avenaceum*	AM12
14	*Fusarium oxysporum*	AM13
15	*Fusarium oxysporum*	AM21
16	*Fusarium equiseti*	AM15
17	*Fusarium equiseti*	AM22
18	*Fusarium tricintum*	AM16
19	*Fusarium semitectum*	AM20
20	*Fusarium scirpi*	AM199
21	*Fusarium solani*	AM203
22	*Aspergillus sp.*	AM31
23	*Aspergillus glaucus*	AM211
24	*Aspergillus nidulans*	AM243
25	*Aspergillus ochraceus*	AM370
26	*Aspergillus candidus*	AM386
27	*Absisia cylindrospora*	AM336
28	*Mortieralla isabellina*	AM212

29	*Trametes versicolor*	AM536
30	*Inonotus radiatus*	AM70
31	*Poria placenta*	AM36
32	*Poria placenta*	AM38
33	*Laetiporus sulphurens*	AM515

Table S2. Strains purchased from the ATCC, KKP, DSMZ, and CBS collection.

No.	Strain name	Strain code
1	*Aspergillus niger*	ATCC 1015
2	*Aspergillus niger*	ATCC 9029
3	*Streptomyces viridosporus*	ATCC 39115
4	*Aspergillus versicolor*	KKP 446
5	*Aspergillus flavus/oryzae*	KKP 3626
6	*Aspergillus flavus*	KKP 3556
7	*Trichoderma harzianum*	KKP 535
8	*Bacillus subtilis*	KKP 3785
9	*Bacillus subtilis*	KKP 3637
10	*Fusarium sp.*	KKP 527
11	*Fusarium oxysporum*	KKP 762
12	*Fusarium oxysporum*	KKP 458
13	*Fusarium oxysporum*	KKP 757
14	*Phanerochaete chrysosporium*	KKP 784
15	*Aspergillus niger*	KKP 44
16	*Aspergillus niger*	KKP 45
17	*Aspergillus niger*	KKP 46
18	*Aspergillus niger*	KKP 422
19	*Aspergillus niger*	KKP 423
20	*Aspergillus niger*	KKP 424
21	*Aspergillus niger*	KKP 606
22	*Aspergillus flavus*	KKP 689
23	*Aspergillus flavus*	KKP 686
24	*Penicillium chrysosporium*	KKP 754
25	*Penicillium chrysosporium*	KKP 784
26	*Penicillium chrysogenum*	KKP 3496
27	*Trichoderma lignorum*	KKP 786
28	*Aspergillus terreus*	DSM 5770
29	*Cordyceps militaris*	DSM 1153
30	*Monascuc ruber*	DSM 62748
31	*Monascus purpureus*	DSM 1379
32	*Penicillium chrysosporium*	DSM 6909
33	*Pseudomonas sp.*	DSM 104485
34	*Pseudomonas sp.*	DSM 27201
35	*Pycnoporus cinnabarinus*	DSM 3022
36	*Pycnoporus cinnabarinus*	DSM 15225
37	*Rhodococcus opacus*	DSM 1069

38	*Streptomyces sannanensis*	DSM 41705
39	*Trametes pubescens*	DSM 9795
40	Phanerochaete chrysosporium	DSM 1547
41	*Trichoderma viride*	DSM 63065
42	*Debaryomyces hansenii*	DSM 70590
43	*Rhodococcus ruber*	DSM 7512
44	*Pseudomonas stutzeri*	DSM 8219
45	*Pycnoporus cinnabarinus*	CBS 311.33
46	*Pycnoporus cinnabarinus*	CBS 374.34
47	*Phanerochaete chrysosporium*	CBS 481.73
48	*Phanerochaete chrysosporium*	CBS 246.84
49	*Mortierella isabellina*	CBS 208.32
50	*Aspergillus niger*	CBS 626.26

Table S3. Strains isolated from various environments.

No.	Strain name	Strain code
1	*Arcopilus aureus*	WPN
2	*Hypoxylon petriniae*	Sb46
3	*Chaetomium globulosum*	j99
4	*Chaetomium subaffine*	SB45
5	*Gliocladiyum rosea*	G2
6	*Heterobasidion annosum*	d22
7	*Rhizosphaera macrospora*	J52
8	*Colletotrichum dematium*	KP2
9	*Trametes versicolor*	d28
10	*Cryptosporidium tarraconensis*	KP1
11	*Trichoderma atroviride*	d43
12	*Trichoderma polyspora*	G3
13	*Penicillium sp.*	SKN07
14	*Penicillium sp.*	SKN014
15	*Penicillium sp.*	L8-5
16	*Penicillium sp.*	L8-1
17	*Penicillium sp.*	L8-19
18	*Penicillium sp.*	L8-7
19	*Penicillium sp.*	L8-9
20	*Penicillium sp.*	L8-18
21	*Fusarium tricinctum*	d48
22	*Fusarium tricinctum*	d61
23	*Fusarium tricinctum*	j106
24	*Fusarium avenaceum*	j111b
25	*Fusarium avenaceum*	d78
26	*Fusarium sporotrichioides*	lg34
27	*Fusarium lateritium*	dokKJ
28	*Fusarium oxysporum*	G6

29	*Fusarium sporotrichioides*	j84
30	*Fusarum culmorum*	KP3 (05.22)
31	*Fusarium oxysporum sansevera*	KP4
32	*Fusarium acuminatum*	S1
33	*Penicillium chrysogenum*	S4
34	*Mucor hiemalis*	W2
35	*Penicillium commune*	W7
36	*Aspergillus versicolor*	TJ1
37	*Trichoderma atroviride*	TRW
38	*Biscogniauxia nummularia*	j56
39	*Penicillium expansum*	dol
40	*Penicillium polonicum*	t8
41	*Penicillium sp.*	nn
42	*Aspergillus niger*	SB47
43	*Cryptosporidium tarraconensis*	C1
44	*Cryptosporidium tarraconensis*	C7
45	*Papulaspora*	G11
46	*Phacidium lacerum*	WPN17/2019
47	*Gliomastix sp.*	lg29
48	*Nemenia serpens*	j37
49	*Paraphaesphaeria neglecta*	d59
50	*Phomopsis velata*	j47
51	*Schizophyllum commune*	d62
52	*Truncatella spartii*	WPN16 L
53	*Monochaeta konsensis*	WPN16 II L
54	*Pycnopeziza sympodialis*	j35
55	N/A	Perenochaeta_3
56	N/A	Perenochaeta_1
57	N/A	Perenochaeta_2
58	N/A	TH1_1
59	N/A	TH2_2
60	N/A	TH5_3
61	N/A	TH3_2
62	N/A	WR4_3
63	N/A	WR3_4
64	N/A	WR3_3
65	N/A	WR3_1
66	N/A	WR3_2
67	N/A	WR4_2
68	N/A	WR2_6

2. Screening process

Cultures were carried out in 250 mL Erlenmayer flasks using 10 g of raw material. After sterilization (121 °C, 15 min) the moisture content was adjusted from 60% by adding sterile deionised water and inoculation (OD_{600} 0.3) and incubated for 144 h at 23 °C.

Culture samples were extracted with a water immiscible organic solvent (ethyl acetate) by shaking, after centrifugation 4 000 rpm, 15 minutes), the organic fraction was dried using anhydrous magnesium sulphate, filtered through vial syringe filters. The evaporated samples were suspended in methanol and used for HPLC analysis. The strains in which vanillin was identified in their extracts were designated for further studies.

Table S4. Results of optimization process through the Box–Behnken design on vanillin biosynthesis in Erlenmeyer flasks from BSG.

Run no.	A	B	C	D	Vanillin content in the extracts [mg/kg d.m. of substrate]						
					Aspergillus sp. AM31	*P. chrysosporium* CBS246.84	*P. chrysosporium* CBS481.73	*A. flavus* KKP3556	*P. cinnabarinus* DSM3022	*P. chrysosporium* DSM6909	*F. culmorum* MUT5855
1	60	35	2	0.2	46	79	78	92	166	107	85
2	60	35	0.5	0.3	70	221	172	137	**204**	132	134
3	70	25	2	0.3	103	128	60	114	61	89	80
4	60	30	2	0.3	65	149	127	155	59	114	178
5	60	30	0.5	0.4	95	**363**	**229**	123	114	**164**	186
6	70	30	0.5	0.3	121	194	183	**218**	139	148	103
7	60	30	0.5	0.2	93	324	181	119	107	139	155
8	50	35	2	0.3	45	116	80	8	124	68	71
9	60	25	2	0.4	119	178	95	107	61	103	134
10	60	30	2	0.3	58	135	112	149	99	132	171
11	60	35	3.5	0.3	25	93	53	81	90	83	73
12	60	30	3.5	0.2	43	85	46	79	70	67	85
13	60	25	2	0.2	99	175	70	88	55	98	123
14	70	35	2	0.3	33	85	55	147	143	78	45
15	70	30	3.5	0.3	29	80	51	136	83	56	56
16	50	30	2	0.4	58	85	97	91	109	73	90
17	60	25	3.5	0.3	64	105	64	83	51	69	95
18	50	25	2	0.3	97	186	85	80	64	65	136
19	70	30	2	0.2	62	107	89	183	113	74	67
20	70	30	2	0.4	66	94	105	194	117	77	78
21	60	35	2	0.4	37	151	90	138	186	104	97
22	50	30	0.5	0.3	85	182	138	116	126	122	146
23	60	25	0.5	0.3	**176**	298	189	129	85	139	**203**
24	60	30	2	0.3	75	163	152	132	91	79	163
25	60	30	3.5	0.4	52	90	75	82	79	65	90
26	60	30	2	0.3	60	197	94	103	132	125	110
27	50	30	3.5	0.3	41	74	50	67	72	48	74
28	50	30	2	0.2	56	111	87	89	103	70	79
29	60	30	2	0.3	77	181	132	99	117	55	165
